# Epigenetic Modifications, and Alterations in Cell Cycle and Apoptosis Pathway in A549 Lung Carcinoma Cell Line upon Exposure to Perfluoroalkyl Substances

**DOI:** 10.3390/toxics8040112

**Published:** 2020-11-23

**Authors:** Musarrat Jabeen, Muhammad Fayyaz, Joseph Irudayaraj

**Affiliations:** 1 Department of Bioengineering, University of Illinois at Urbana-Champaign, Urbana, IL 61801, USA; mjabeen2@illinois.edu (M.J.); mfayyaz2@illinois.edu (M.F.); 2 Biomedical Research Centre (BRC) at Mills Breast Cancer Institute, Carle Foundation Hospital, Urbana, IL 61801, USA; 3 Cancer Centre at Illinois (CCIL), University of Illinois at Urbana-Champaign, Urbana, IL 61801, USA; 4 Nick Holonyak Micro and Nanotechnology Laboratory, University of Illinois at Urbana-Champaign, Urbana, IL 61801, USA; 5 Carl R. Woese Institute of Genomic Biology, University of Illinois, Urbana-Champaign, Urbana, IL 61801, USA

**Keywords:** PFAS: GenX, PFOA, PFOS, epigenetics, cell proliferation cycle, apoptosis, lung cancer

## Abstract

Per- and polyfluoroalkyl substances (PFAS) are a group of human-made compounds with strong C-F bonds, and have been used in various manufacturing industries for decades. PFAS have been reported to deleterious effect on human health, which has led to studies identifying the possible toxicity and toxicity routes of these compounds. We report that these compounds have the potential to cause epigenetic modifications, and to induce dysregulation in the cell proliferation cycle as well as apoptosis in A549 lung cancer cells when exposed to 10-, 200- and 400 μM concentrations of each compound. Our studies show that exposure to perfluorooctanoic acid (PFOA) and perfluorooctane sulfonic acid (PFOS) may cause hypomethylation in the epigenome, but changes in the epigenetic makeup are not evident upon exposure to GenX. We establish that exposure to lower doses of these compounds causes the cells’ balance to shift to cell proliferation, whereas exposure to higher concentrations shifts the balance more towards apoptosis. Furthermore, the apoptosis pathway upon exposure to GenX, PFOA, and PFOS has also been identified. Our findings suggest that exposure to any of these compounds may have profound effects in patients with pre-existing lung conditions or could trigger lung cancinogenesis.

## 1. Introduction

Per- and polyfluoroalkyl substances (PFAS) are a group of synthetic perfluoroalkyl compounds, including short chain (4–7 carbon chain length) and long-chain (8–12 carbon chain length) perfluorinated carboxylic acids (PFCAs), perfluorinated sulphonic acids (PFSAs) and perfluoroalkyl ether carboxylic acids (PFECAs). Their surfactant properties make them useful in many industries such as surface coatings, cosmetics, carpets and rugs, protectant formulations, firefighting foams [1,2] and non-stick coatings on cookware [3]. Owing to their strong carbon–fluorine bonds, perfluoroalkyl substances are resistant to hydrolysis, biodegradation, direct photolysis, and photooxidation [2,4], making them very stable and resistant to biodegradation. Moreover, they have very low volatility due to their ionic nature [1,5,6] and, therefore, persist in soil and water [5] and possess the ability to leach into the soil, contaminating the groundwater sources [7]. Perfluoroalkyl substances have been detected in food, groundwater, ocean water, surface water, dust, indoor air, outdoor air, and in drinking water [8], implying that exposure to humans may occur through all these sources. PFAS are readily absorbed following any route of exposure and are not readily metabolized in humans or laboratory animals, making them highly bioavailable. The longer the chain length, the higher the bioaccumulation potential of the PFAS [9,10,11]. Depending on the hydrophobic and lipophobic nature of compounds, different PFAS tend to accumulate in different organs, with lungs being the most bioaccumulative organ [12,13]. The highest concentrations of perfluorooctanoic acid (PFOA) and perfluorooctane sulfonic acid (PFOS) were found in bone and liver, respectively [14], and that of hexafluoropropylene oxide-dimer acid (HFPO-DA), referred to as GenX are found in the blood and liver [15,16].

Perfluoroalkyl substances have been detected in blood serum [17,18,19,20,21], umbilical cord blood [22,23,24,25,26] and breast milk [27,28] of general and highly exposed population. Adult uptake doses estimated for low, medium, and high-exposure scenarios were approximately 7, 15, and 30 ng/kg of body weight/day, respectively, for PFOS and about 0.4, 2.5, and 41–47 ng/kg of body weight/day, respectively, for PFOA. Exposures estimated for infants, toddlers, and children under 12 years were significantly higher [29,30]. Considering these doses of daily uptake, the estimated elimination half-lives of these compounds in humans are 3.8 years and 5.4 years for PFOA and PFOS, respectively, [20] with relatively shorter elimination half-lives in animals such as non-human primates [31,32,33], rats [33,34] and mice [33,35]. The half-life of HFPO-DA in humans is not known. However, elimination half-life in laboratory animals ranges from one to several days [36].

Studies have been done to establish the toxicity of PFASs in humans through epidemiological studies in humans, and in the laboratory animals considering oral exposure as the primary route, with developmental, reproductive [37,38,39], hepatic [40,41,42,43], thyroid disease [44] and immunological effects [45,46,47,48] being the most common study endpoints. Evidence of the carcinogenic potential of PFAS in highly exposed individuals [8,49,50,51,52] also exists. Since these compounds have been reported to be accumulated in the most substantial quantity in lungs [8], we chose to study the effects of exposure to GenX, PFOA, and PFOS in a human non-small cell lung carcinoma cell line (A549). Recently, our lab has published work on the potential of these compounds to cause epigenetic alterations [53,54,55,56]. Epigenetic alterations have been widely studied as the stable genetic modifications, capable of being transferred across generations with no changes in DNA sequence, and have been known to play a critical role in development [57,58,59] in healthy as well as diseased states [60,61,62]. Modifications constitute DNA methylation, non-coding RNAs-based silencing, and histone modifications. DNA methylation and histone modifications may interact with each other in the regulation of chromatin [63]. This study focuses on the DNA methylation-induced epigenetic alterations in response to PFASs exposure. DNA methylation is characterized by the addition of the methyl (-CH_3_) group to the cytosine guanine dinucleotides (CpG) sites of DNA, which is highly organized and regulated by two groups of enzymes: DNA methyltransferases (DNMTs) [64] and Ten-eleven translocation (TETs) [65] enzymes. The human genome possesses five DNA methyltransferases: DNMT1, DNMT3a, and DNMT3b with catalytic activity, and DNMT2 and DNMT3L with non-catalytic activity [66]. In this study, we investigate the changes in mRNA expression for the catalytic DNMTs and TETs upon exposure to PFASs.

We also study the effect of PFAS on apoptosis and cell cycle regulation. The precise orchestration of apoptosis is critical to the development of organisms, and the adequate removal of damaged or cancerous cells, making apoptosis a highly programmed cell death [67]. PFASs have been implicated in inducing apoptosis in cells through the production of reactive oxygen species (ROS) and oxidative stress [68,69]. Precise regulation of apoptotic cell death involves an interplay of apoptotic and anti-apoptotic proteins. Here, we examine the mRNA expression of genes for apoptotic and anti-apoptotic proteins as well as caspases, which are the proteases executing apoptosis. Moreover, we investigate the changes in the cell cycle regulator genes to determine any dysregulation in cell proliferation, which is the first and foremost step in the evolution of cancer [70].

We used human non-small cell lung carcinoma cell line (A549), since most of the PFAS have the highest potential to accumulate in the lungs. We attempt to establish the effects on epigenetic alterations, and on the interplay between dysregulation of cell proliferation cycle and apoptosis in the lungs in response to the exposure to different concentrations of GenX, PFOA, and PFOS. Based on our observations, we chose a concentration of each of these compounds to quantify the intracellular accumulation through Ultra performance liquid chromatography-Mass spectrometry (UPLC-MS) and imaged through Hyperspectral dark-field microscopy (HS-DFM).

## 2. Materials and Methods

### 2.1. Dosing Solutions

Three perfluoroalkyl compounds were used in the experiments: perfluorooctanoic acid (PFOA) of 95% purity (Cat. no. 171468) was purchased from Sigma-Aldrich, USA, perfluorooctane sulphonic acid (PFOS) with 97% purity (Cat. no. 6164-3-08) and GenX (also called 2,3,3,3-tetrafluoro-2-(heptafluoropropoxy) propanoic acid/Undecafluoro-2-methyl-3-oxahexanoic acid) of 97% purity (CAS no. 13252-13-6), were purchased from Synquest Laboratories Inc., USA. All of the compounds were constituted in Dimethyl Sulfoxide (DMSO, Cat no. 25-950-CQC, Corning Inc., New York, NY, USA) to make one molar stock solution. The stock solutions were then spiked into the cell culture medium to make 10 to 1000 μM working concentrations such that the final concentration of DMSO in the treatment medium and vehicle control was ≤0.1%. As discussed below with the cell viability assay, 0.1% of DMSO is non-toxic to cells. 

### 2.2. Cell Culture

The human non-small cell lung carcinoma cell line (A549 ATCC^®^ CCL-185^™^) was purchased from the American Type Culture Collection, USA. Cell cultures were maintained in T-25 flasks in F-12K medium (Cat. no. ATCC^®^ 30-2004^™^) supplemented with 10% Fetal bovine serum (Cat. no. 16140071, Gibco^™^) and 1% Penicillin–Streptomycin solution (Cat. no. ATCC^®^ 30-2300^™^) at the density of 6 × 10^3^ to 6 × 10^4^ cells/cm^2^ at 37 °C in the humidified incubator (Forma™ Series II Water-Jacketed CO_2_ Incubators, Thermo Scientific™). For exposure experiments, cells were seeded at a density of 5 × 10^3^ cells/cm^2^ in T-25 flasks. At 30% confluence, the culture medium was replaced with the compound-containing medium at 10, 200 and 400 μM concentrations, and vehicle-control containing 0.04% DMSO-medium (equal to the DMSO in the highest concentration of compound used). Three individual replications were performed for all of the concentrations of each compound. After 48 h of treatment, cells were trypsinized, harvested and the pellets were stored at −80 °C for downstream experiments.

### 2.3. Cell Viability Assay

The cell viability was determined using an 3-(4,5-Dimethylthiazol-2-yl)-2,5-Diphenyltetrazolium Bromide (MTT) assay as a function of redox potential. The yellow-colored water-soluble MTT reagent is converted to insoluble purple-colored formazan crystals, which are then solubilized, and optical density was determined as a measure of actively metabolizing cells. The MTT reagent (Cat. no. M6494) was purchased from Thermo Fisher Scientific, USA, and dissolved in 1X phosphate-buffered saline (PBS) (Cat. no. 21-040-CV, Corning Inc., Corning, NY, USA) at a concentration of 5 mg/mL and filter-sterilized. The reagent was freshly prepared for every experiment. The A549 cells were seeded in a 96-well plate at a density of 7000 cells/well and allowed to adhere for 24 h. The cell culture medium was then replaced with vehicle-control (DMSO-medium) and the compound-containing medium at concentrations ranging from 10 to 1000 μM, and cells were incubated. After 24 and 48 h, 20 µL freshly prepared 5 mg/mL MTT reagent was added to each well and incubated for 3.5 h, the medium was then removed carefully and 150 μL acidified 0.1% IGEPAL^®^ CA-630 (Cat. no. I8896, Sigma-Aldrich, St. Louis, MO, USA) in molecular biology grade Isopropanol (Cat. no. BP2618500, Fisher Scientific, Waltham, MA, USA) was added to each well. The plates were wrapped in aluminum foil and placed on an orbital shaker for 20 min to assure complete solubilization of the formazan crystals. The optical density was recorded at 570 nm with a reference at 620 nm using a microplate reader. For all the concentrations, three replications were done on each plate and results were averaged; the experiment was repeated two more times for all three compounds at different time-points (n = 3). 

### 2.4. Isolation and Quantification of RNA and cDNA Synthesis

Total ribonucleic acid (RNA) was isolated from the −80 °C stored cell pellets using TRIzol™ Reagent (Cat. no. 15596018, Thermo Fisher Scientific, USA) according to manufacturer’s protocol, followed by treatment with DNase and purification using RNeasy mini kit (Qiagen Inc., Germantown, MD, USA). The isolated RNA was quantified with purity assessment using NanoDrop™ One Microvolume UV-Vis Spectrophotometer (Thermo Fisher, Waltham, MA, USA). The High-Capacity cDNA Reverse Transcription Kit (Cat. no. 4368814, Applied Biosystems, Foster City, CA, USA) was then used to synthesize complementary DNA (cDNA) from the isolated RNA and was confirmed by gel electrophoresis. 

### 2.5. Quantitative Real-Time Polymerase Chain Reaction (qRT-PCR)

The quantitative real-time polymerase chain reaction (qRT-PCR) was performed a using StepOnePlus™ Real-Time PCR System (v 2.0 Applied Biosystems, USA) for the DNA methylases, TETs, genes for apoptotic and anti-apoptotic proteins, caspases and cell cycle regulation. The reaction was set up in a 96-well reaction plate in triplicate using 20 μL of PowerUp SYBR Green Master Mix (Applied Biosystems, USA) and 5 μL cDNA. NCBI BLAST was used to synthesize gene-specific forward and reverse primers, listed in Table S1; for epigenetic analysis (Table S1a), for cell cycle proliferation study (Table S1b), and for apoptosis and pathway studies (Table S1c); the primers were purchased from Integrated DNA Technologies, Inc., Coralville, IA, USA. Three independent experiments were done, the ΔΔCt method was used for data analysis, and results are presented as an expression of the gene relative to Glyceraldehyde 3-phosphate dehydrogenase (GAPDH).

### 2.6. Intracellular Concentration Determination by UPLC-MS Analysis

For concentration analysis, we used a previously published method with a slight modification [13,71]. Briefly, the cells were seeded in 25 cm^2^ flask in F-12K medium supplemented with 10% Fetal Bovine Serum (FBS) and 1% penicillin/streptomycin. After 24 h, the cell culture medium was replaced with 400 μM GenX, PFOA and PFOS, along with vehicle control as previously described [69,70]. Following 48 h of treatment, the cells were harvested and washed three times with ice-cold PBS. The cells were then lysed in 0.5% TritonX-100 (Sigma) in deionized water at −20 °C. The lysates were then thawed, followed by centrifugation for 10 min at 18,000× *g* 4 °C. The supernatant was collected and stored at −20 °C until analysis. The protocol for UPLC-MS analysis was developed in the Mass spectrometry laboratory, School of Chemical Sciences at Illinois. The UPLC-MS (Waters Synapt G2) high resolution negative mode-based method with an autosampler was used to quantify the intracellular concentrations of PFOA, PFOS and GenX. The mobile phase consisted of 5 mM Ammonium acetate as a solvent A and Acetonitrile as solvent B. The R^2^ of calibration curve for PFOA and PFOS, was 0.9959 and 0.9986, respectively. The lower limits of quantification used for PFOA and PFOS were 0.097 µM and PFOS 6.25 µM, respectively. Unfortunately, we could not quantify GenX because the calibration curve did not fit well after several repetitive experiments. This is perhaps because GenX could not be ionized easily.

### 2.7. Hyperspectral Dark-Field Microscopy (HS-DFM)

For imaging, the cells were cultured on positively charged slides and allowed to adhere. After 24 h, the medium was replaced with the one containing 400 μM GenX, PFOA, and PFOS, separately, and incubated for 2 h at 37 °C. The slides were then washed with phosphate-buffered saline (PBS) and cells were fixed with 4% paraformaldehyde. The method we used for identification was developed by our group and is published elsewhere [72,73]; it uses an enhanced dark field illumination system (CytoViva, Auburn, AL, USA). The cells grown on positively charged slides without any compound administration were scanned and captured as a control image. The spectral data were analyzed by using the CytoViva software program (ENVI 4.8 and ITT Visual Information Solutions). The processing of image and data interpretation included steps that are essential for creating spectral libraries. The spectral libraries were collected by defining a region of interest (ROI) from the scanned specimen. When the required specific spectral libraries were recognized, they were kept in a spectral library folder by the CytoViva ENVI software for the following spectral mapping of the hyperspectral images of other specimens. Each spectrum involved in the library was collected from a single pixel imaged with a 40X objective. Eventually, a standard Spectral Angle Mapper (SAM), was applied to estimate the resemblance between the pixels of the image and the spectral library pixels saved in the CytoViva ENVI software folder.

### 2.8. Statistical Analysis

The statistical analysis for all the experiments was done using GraphPad Software version 8.3.

## 3. Results

### 3.1. Cytotoxicity Estimation

**Figure 1:**
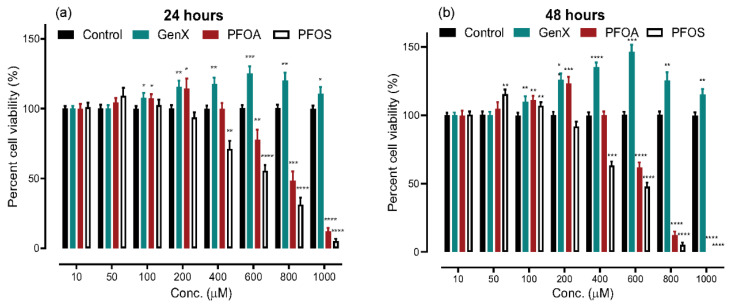
Cytotoxicity evaluation of A549 cells exposed to GenX, perfluorooctanoic acid (PFOA) and perfluorooctane sulfonic acid (PFOS) through 3-(4,5-Dimethylthiazol-2-yl)-2,5-Diphenyltetrazolium Bromide (MTT) assay. (**a**) 24-h exposure time. (**b**) 48-h exposure time. We observed a similar pattern from cell viability analysis, but an exaggerated response in 48-h exposed cells. GenX increases cellular proliferation beyond 50 μM concentration. PFOA and PFOS show a characteristic significant increase in proliferation at 100- to 200 μM concentration, but a decrease in viability beyond 200 μM (PFOS > PFOA). The data are given as the mean ± S.D. for three independent experiments (n = 3) (* = *p* < 0.05, ** = *p* < 0.005, *** = *p* < 0.001, **** = *p* < 0.0001).

We used an MTT assay to determine the cytotoxic potential of different concentrations of the three compounds: GenX, PFOA, and PFOS after 24- and 48-h exposure. The results indicate that PFOA and PFOS have higher cytotoxic potential than GenX at any given concentration, with PFOS being more toxic than PFOA, based on the relative percentage of cell viability, given in Figure 1 as a percentage of the control values for the cells grown concurrently in the vehicle-control. The results were exaggerated when the exposure time was increased from 24 h to 48 h, but exhibited the same pattern. Upon exposure to GenX, the cells showed significantly increased proliferation starting at 100 μM concentration. A similar increase in cellular proliferation was also noted in 100- and 200 μM concentrations of PFOA and PFOS exposure; however, we noted a significant decrease in cell viability at and beyond 600 μM PFOA and 400 μM PFOS exposure. In order to explain the increased proliferation at the lower concentrations, we opted to check for the mRNA expression of genes for cell cycle proliferation at 10, 200 and 400 μM concentrations and used the same concentrations throughout to keep it consistent.

### 3.2. Exposure to PFAS Alters the mRNA Expression of DNA Methylation Regulators

**Figure 2:**
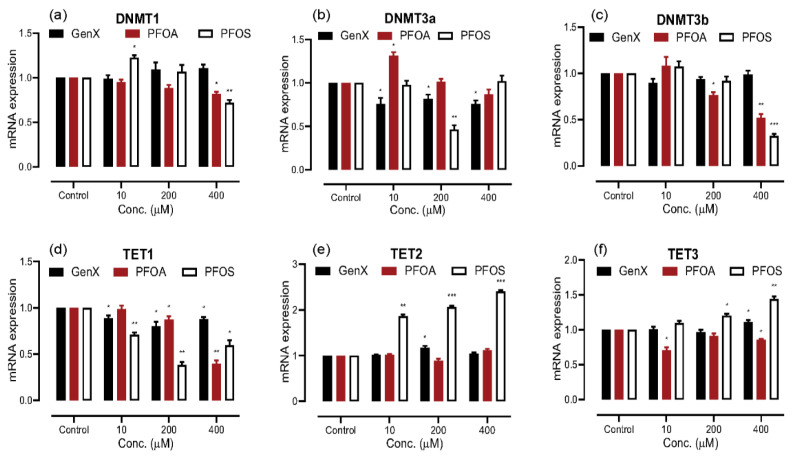
Epigenetic analysis of A549 cells exposed to GenX, PFOA and PFOS for 48 h, through quantitative real-time polymerase chain reaction (qRT-PCR). (**a**) and (**c**) DNA methyltransferases (DNMT)1 and DNMT3b mRNA expression significantly decreased upon exposure to higher concentration of PFOA and PFOS. (**b**) DNMT3b mRNA expression did not show any particular trend. (**d**) Dose-related statistically significant decrease in mRNA expression of Ten-eleven translocation (TET)1. (**e**) Dose-related statistically significant increase in mRNA expression of TET2 upon PFOS exposure. (**f**) An increase in mRNA expression of TET3 when exposed to higher doses of PFOA and PFOS. The data are given as the mean ± S.D. for three independent experiments (n = 3) (* = *p* < 0.05, ** = *p* < 0.005, *** = *p* < 0.001).

DNA methylation is one of the three epigenetic alteration mechanisms, which is closely regulated by DNA methyltransferases and Ten-Eleven translocation (TETs) enzymes. We used the cDNA from the RNA isolated from cells exposed to the three different concentrations of the three compounds to determine mRNA expression levels of six major regulators of DNA methylation: DNMT1, DNMT3a, DNMT3b, TET1, TET2 and TET3, through qRT-PCR (Figure 2). The mRNA expression for DNMT1 and DNMT3b significantly decreased upon exposure to 200- and 400 μM concentrations of PFOA and 400 μM PFOS; we also noticed a significant increase in the expression of DNMT1 at 10 μM PFOS exposure. However, no significant change in DNMT1 and DNMT3b expression was observed with GenX exposure at any concentration; a significant decrease in the mRNA expression for DNMT3a at all the concentrations tested was noted. The mRNA expression for DNMT3a upon PFOA and PFOS exposure did not follow any particular pattern; 10 μM PFOA exposure showed an increase, and 200 μM PFOS exposure showed a decrease in the expression. All three compounds caused a significant decrease in the mRNA expression levels of TET1 at all of the concentrations tested. The PFOS exposure exhibited a highly significant increase in the mRNA expression for TET2 at all the concentrations tested, whereas no significant change was observed for TET2 with the other two compounds, except for an increase at 200 μM GenX exposure. A significant increase was observed in the mRNA expression for TET3 when exposed to 400 μM PFOA and 200- and 400 μM PFOS, with a decrease noted for GenX exposure at all concentrations tested. 

### 3.3. PFAS Exposure Causes Dysregulation in the Cell Cycle Proliferation Genes

**Figure 3:**
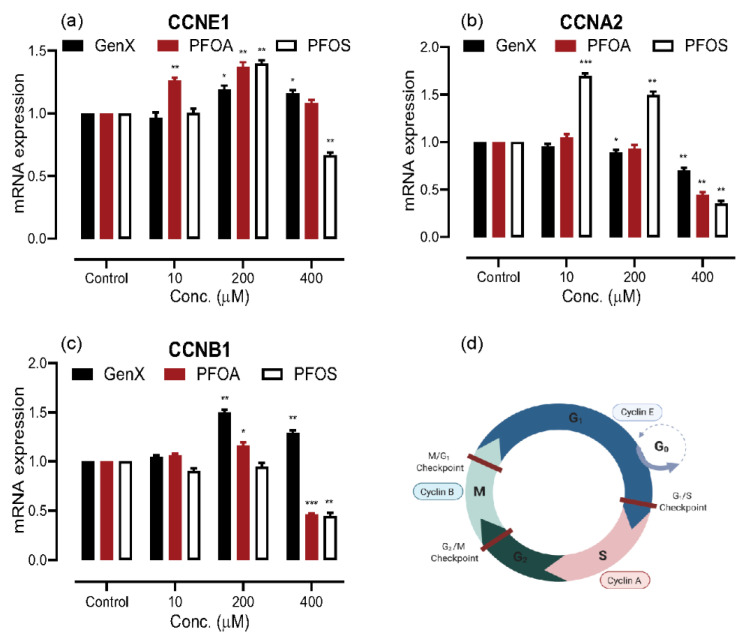
Analysis of cell proliferation cycle genes in A549 cells upon exposure to 10-, 200- and 400 μM concentrations of GenX, PFOA and PFOS through quantitative real-time polymerase chain reaction (qRT-PCR). (**a**) mRNA expression of cyclin (CCN)E1 shows a trend of an increase at lower concentration of all the compounds, followed by a decrease in expression as the concentration of compounds increased. (**b**) CCNA2 mRNA expression is increased upon exposure to low concentrations of PFOA and PFOS, and decreased very significantly upon exposure to 400 μM. (**c**) mRNA expression of CCNB1 increased at lower doses (more with GenX), following a very significant decrease in expression at 400 μM of PFOA and PFOS. (**d**) Normal cell cycle and the respective role of cyclins. The data are presented as the mean ± S.D. for three independent experiments (n = 3) (* = *p* < 0.05, ** = *p* < 0.005, *** = *p* < 0.001).

The cell cycle in any eukaryotic cell comprises four stages: Gap 1 (G1 phase), Synthesis (S phase), Gap 2 (G2 phase), and mitosis (M phase). Cell cycle is strictly regulated by cyclin-dependent kinases (CDKs), which are meticulously controlled by cyclins. We performed qRT-PCR to evaluate changes in mRNA levels of three cyclins, responsible for regulating the CDKs (Figure 3). The mRNA expression of cyclin E1 (CCNE1) was observed to be increased at 10- and 200 μM exposure, showing the trend to drop at 400 μM exposure for all of the three compounds. The PFOS exposure presented a similar trend in cyclin A2 (CCNA2) mRNA expression as with CCNE1; however, the CCNA2 mRNA expression very significantly decreased upon exposure to 400 μM in all the compounds tested. The GenX and PFOA exposure showed a similar trend in cyclin B1 (CCNB1) as in CCNE1, the PFOS exposure caused a decreased expression at all the concentrations tested. Moreover, 400 μM exposure to PFOA and PFOS decreased the expression very significantly.

### 3.4. Induction of Apoptosis on PFAS Exposure

**Figure 4:**
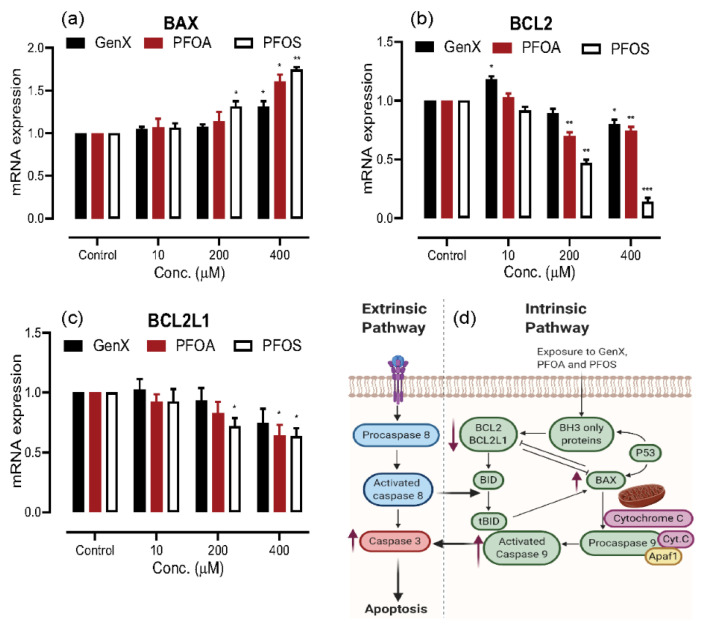
Determination of apoptosis induction in A549 cells upon exposure to GenX, PFOA and PFOS through quantitative real-time polymerase chain reaction (qRT-PCR). (**a**) Dose-dependent increase in mRNA expression levels of pro-apoptotic gene BAX, for all the compounds. (**b**) and (**c**) Dose-dependent decrease in the mRNA expression of anti-apoptotic genes BCL2 and BCL2L1, more significant with PFOS exposure. (**d**) The apoptosis pathway, red arrows indicate the change in expression in response to polyfluoroalkyl substances (PFAS)s. The data are given as the mean ± S.D. for three independent experiments (n = 3) (* = *p* < 0.05, ** = *p* < 0.005, *** = *p* < 0.001).

The process of apoptosis is very critical to the healthy development of tissues and to have the body rid of abnormal cells, which is highly regulated by a group of pro-apoptotic and anti-apoptotic proteins. Figure 4 shows the results of mRNA expression of genes for one pro-apoptotic (BAX) and two anti-apoptotic proteins (BCL-2 and BCL2L1) in response to exposure to GenX, PFOA, and PFOS. We observed a dose-dependent increase in the mRNA expression of pro-apoptotic gene BAX and a similar dose-related decrease in the anti-apoptotic genes BCL-2 and BCL2L1, being more pronounced in PFOS > PFOA > GenX. 

### 3.5. PFAS Induced Apoptosis through the Intrinsic Pathway

**Figure 5:**
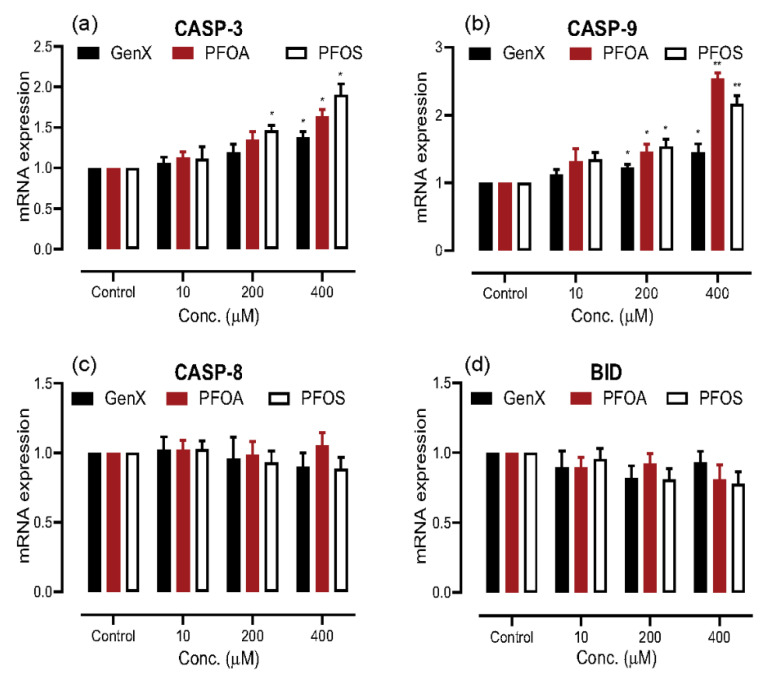
Identification of the apoptosis pathway taken by A549 cells on exposure to GenX, PFOA and PFOS. (**a**,**b**) Dose-related increase in the mRNA expression of caspase (CASP)-3 and CASP-9, respectively, for all the compounds. (**c**,**d**) mRNA expression of CASP-8 and BH3 interacting-domain death agonist (BID), respectively, showing no statistically significant change on exposure to any concentration of all the compounds. The results are presented as the mean ± S.D. for three independent experiments (n = 3) (* =*p* < 0.05, ** = *p* < 0.005)**.**

Our results for pro-apoptotic and anti-apoptotic genes expression analysis indicated the induction of apoptosis, which led us to identify the apoptosis pathway the cells may take in response to exposure to these compounds. The cells destined for apoptosis can take either an intrinsic or an extrinsic pathway. In order to identify that, we used qRT-PCR to see any alterations in the mRNA expression of the genes for three cysteine-aspartases (Caspases), presented in Figure 5a–c. We observed a dose-dependent increase in mRNA expression for caspase 3 (CASP3) and caspase 9 (CASP9) for all the compounds, with CASP9 mRNA expression increasing two- to three-fold when the concentration was increased to 400 μM for PFOA and PFOS. However, statistically significant change was not observed in the expression levels of caspase 8 (CASP8) for any compound at all the concentrations tested. Further, we checked if there was any statistically significant change in the mRNA expression of BH3 interacting-domain death agonist (BID), which could relate to the extrinsic and intrinsic pathways, but found no significant change at any concentration for all the compounds (Figure 5d).

### 3.6. Intracellular Concentration Analysis

**Figure 6:**
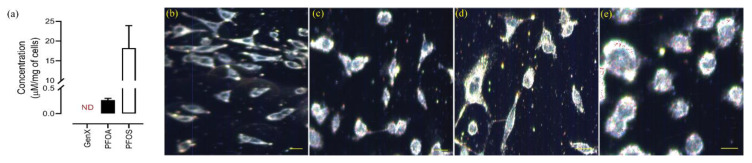
Intracellular levels of PFASs using UPLC-MS, and imaging of cells with Hyperspectral dark-field microscopy (HS-DFM) imaging. (**a**) The PFOS has the highest potential to accumulate inside A549 cells, the GenX was not detected. The data are given as the mean ± S.D. for three independent experiments. ND—not detected. (n = 3) (**b**) Hyperspectral images of A549 cells for control and (**c**–**e**) exposed to GenX, PFOA and PFOS, respectively. The red dots inside the cells denote the compound taken up by the cell, identified by Spectral angular mapping. Scale bar: 10 μm.

Since we observed maximum effects when exposed to 400 μM concentration of compounds, we chose to quantify the intracellular accumulation of these compounds at this concentration. The intracellular concentration of PFOA and PFOS was detected using UPLC-MS (Waters Synapt G2). The results, given in Figure 6a, revealed that the intracellular concentration of PFOA and PFOS detected was 0.266 ± 0.353 µM and 18.24 ± 3.370 µM per milligram of cells, respectively. Our method could not detect any GenX in the samples. This could be due to undetectable quantity of GenX accumulating inside the cells or incomplete ionization of GenX. All the experiments were done in triplicate and results were averaged (n = 3).

### 3.7. Cellular Accumulation of PFAS evaluated by Hyperspectral Dark-Field Microscopic (HS-DFM) Imaging

We chose the same concentration for all the compounds that we had used for concentration analysis for imaging the intracellular accumulation. The identification of each compound uptake by A549 cell line was carried out using the Hyperspectral Dark-Field Microscopy technique. This non-destructive technique provides qualitative information on intracellular biodistribution of particles and allows the determination of accumulation patterns. For our experiments, the A549 cell line was incubated for 2 h with each compound at 37 °C and with 5% carbon dioxide. The cells were fixed on positively charged slides and imaged with a hyperspectral dark-field microscope (CytoViva, Auburn, AL, USA) as shown in Figure 6b–e for the Control, GenX, PFOA, and PFOS, respectively. To identify the compound intracellularly, we developed a spectral library for each compound and saved them in the memory folder; Figure S1 shows the spectral libraries for GenX, PFOA, and PFOS, respectively, along with the images of the compounds used to build the libraries. The spectra were then filtered with each image using a control image as a blank for the identification of the compound in the exposed cells with the (ENVI 4.8 software, CytoViva, Auburn, AL, USA) Spectral Angle Mapper tool. After mapping, particle aggregates uptake by the cells can be performed with Image pro premier 9.0 machine learning tool. The experiments were done at three independent intervals and the results were averaged. The average proportion of GenX, PFOA, and PFOS aggregates taken up by cells after two hours of exposure was the maximum for PFOS, followed by PFOA, and GenX as evidenced from intensity and spectral mapping (n = 3). Our results for compound uptake by cells were in close agreement with the concentration analysis by UPLC-MS, reinforcing that the uptake of PFOS > PFOA > GenX. However, we were not able to quantify GenX through UPLC-MS.

## 4. Discussion

PFAS, the synthetic perfluoroalkyl compounds, have been used for decades due to their unique properties and stability in non-stick coatings [1], fire-fighting foams [3] and many other industries. However, recently, the concerns arose whether these compounds are safe for human beings or not, resulting in an immense interest on the toxicity of this compound. PFAS have been implicated in causing dysregulation in almost all of the organ systems [37,43,48,53,54], causing renal and testicular cancers [8]. Since PFAS have been reported to accumulate in the lungs, we chose to work on a lung carcinoma cell-line (A549) in this study.

Epigenetic modifications have been reported to play a critical role in the development of an organism as well as in the maintenance of healthy tissue [57,58,60,62], and are of three types: DNA methylation, histone modifications and, non-coding RNA-based silencing. Recently, the role of epigenetics in toxicology has opened up a new realm for environmental toxicology [74,75,76]. This work is focused on DNA methylation, which is regulated by DNA methyltransferases (DNMTs) and Ten-eleven translocation (TETs) enzymes. Of DNA methyltransferases, three enzymes possess catalytic activity in humans, DNMT1, DNMT3a and DNMT3b; the former is the maintenance enzyme that methylates the hemi-methylated DNA, whereas the latter two are the de novo enzymes which catalyze the formation of 5-methylcytosine (5-mC), thus establishing the new methylated CpG sites. Ten-eleven translocation (TETs) family of enzymes play a crucial role in the demethylation, by catalyzing the hydroxylation of 5-methylcytosine (5-mC) to 5-hydroxymethylcytosine (5-hmC), 5-hmC to 5-formylcytosine (5-fC) and 5-fC to 5-carboxylcytosine (5-caC). Thus, DNA methyltransferases and Ten-eleven translocation enzymes play a coordinating role in modifying the epigenome [77]. Our results for gene expression analysis for DNMTs and TETs indicated decreased expression of DNMT1 and DNMT3b on exposure to higher doses (200- and 400 μM) of PFOA and PFOS, and increased expression of TET2 on PFOS exposure, and TET3 at higher doses (200- and 400 μM) of PFOA and PFOS. In general, the effect of GenX was not evident in causing any change in the epigenome at the concentrations tested, whereas PFOA and PFOS may cause hypomethylation in the A549 cell-line, more so at higher doses, as indicated by decreased DNMTs and increased expression of TETs. Comparable *in vitro* studies in hepatocellular carcinoma (HepG2) and breast cancer (MCF7) cell-lines [56] and in *in vivo* studies with mouse liver and kidney tissues [54,55] have been reported.

Our results from the cytotoxic evaluation through 3-(4,5-Dimethylthiazol-2-yl)-2,5-Diphenyltetrazolium Bromide (MTT) assay indicated an increase in the cellular proliferation at all the concentrations of GenX exposure. Surprisingly, we noticed an increase in proliferation at 100- to 200 μM of PFOA and PFOS, and a dose-dependent decrease beyond this concentration. To explain this proliferation, we evaluated the mRNA expression levels for the cell proliferation genes upon exposure to the three concentrations of each compound. The highly regulated cellular proliferation is crucial for the development and maintenance of tissues’ and any dysregulation that can potentially lead to carcinogenesis [70,78]. The cell cycle has four phases in a eukaryotic cell, and each of the phases is regulated by cyclin-dependent kinases (CDKs) coupled to the cyclins. The levels of three cyclins, CCNE1, CCNA2, and CCNB1, are tightly synchronized with the progression of the cell cycle. The level of cyclin E (CCNE1) is maximum in the G1 phase, and forms a complex with CDK, which then drives the cell through the G1 phase, preparing it for S-phase. Similarly, the levels of cyclin A (CCNA2) and cyclin B (CCNB1) peak in the S-phase and M-phase, respectively, form complexes with CDKs, and drive the cells through the G2/M and mitotic growth checkpoints. In essence, CCNE1 is noted as G1/S cyclin, CCNA2 as S cyclin and, CCNB1 as M cyclin. In general, our results indicated an increase in the expression of the G1/S and S cyclins at lower doses (10- and 200 μM), with a very significant decrease at 400 μM exposure concentration. However, the changes observed were less significant in GenX and more pronounced with PFOS exposure, which seemed to decrease the expression of M cyclin at all the concentrations tested. From our studies we note that exposure to lower concentrations (10- and 200 μM) of GenX and PFOA may cause increased expression of all three cyclins, thus inducing cellular proliferation, while the higher dose of 400 μM PFOA may reduce the expression of all three cyclins, thus inhibiting cellular proliferation. On the contrary, PFOS exposure at lower doses of 10- and 200 μM increased the expression of only two cyclins G1/S and S cyclin but not the M cyclin, which shows a decreased expression at all the concentrations of PFOS exposure. The 400 μM PFOS gives similar results as of PFOA. This may project successful growth and DNA replication (S) phases but growth restriction at the mitotic checkpoint in response to lower dose exposure to PFOS, whereas, lower dose exposure to GenX and PFOA does allow successful completion of the cell cycle, with 400 μM exposure of PFOA and PFOS which seem to restrict the growth checkpoints in a cell cycle. Our results are comparable to another recent *in vivo* study for PFOA exposure in mice [55].

The altered expression of cell cycle regulator genes upon PFAS exposure prompted us to check for the expression of pro-apoptotic and anti-apoptotic genes since healthy tissue development and maintenance requires a very precise balance of cell proliferation and apoptosis [70]. We checked the mRNA expression of the pro-apoptotic and anti-apoptotic genes, the interplay of which determines the process of apoptosis. Our results identified a classic dose-dependent increase in BAX (pro-apoptotic) expression, with a dose-dependent decrease in the BCL-2 and BCL2L1 (anti-apoptotic) expression, more pronounced with PFOS exposure. Similar results have been published previously establishing the role of these compounds in inducing apoptosis through oxidative stress and reactive oxygen species [55,68,69]. Surprisingly, we noticed an inverse relationship between the expression of cell proliferation genes and apoptosis genes. Therefore, we deduced that low dose (10- and 200 μM) exposure of A549 cell-line to these compounds caused the cells to switch more towards cell proliferation, whereas a higher dose (400 μM) exposure caused the balance to shift more towards apoptosis.

Furthermore, we identified the apoptosis pathway activated by these compounds with mRNA expression levels for the three main cysteine aspartases (caspases), which are protease enzymes responsible as executioners of apoptotic cell death [79]. The cell can take either an intrinsic or an extrinsic pathway of apoptosis, depending on the stimulus. Caspase 9 (CASP9) gets activated during the intrinsic pathway, whereas caspase 8 (CASP8) is activated during the extrinsic pathway of apoptosis. The caspase 3 (CASP3) is the final executor of apoptosis by both the pathways. The extrinsic and intrinsic pathways can communicate through BH3 interacting-domain death agonist (BID). We observed a dose-dependent increase in the expression of CASP9 and CASP3, with no significant change in the expression of CASP8 and BID. There was a two to three-fold increase in expression of CASP9 upon exposure to a higher dose (400 μM) of PFOA and PFOS, which indicated a highly significant activation of the intrinsic pathway of apoptosis. We concluded that exposure to PFASs might cause the activation of the intrinsic pathway (CASP9–CASP3 axis) of apoptosis, similar to the previously published studies for zebrafish liver (ZFL) cell line and A549 PFOS exposure [68,80], and there is no communication between the two pathways since we did not observe any statistically significant change in the mRNA expression of BID. The increased expression of CASP3 in non-small cell lung carcinoma in a clinical study has been reported to be related to the poor prognosis of patients [81]. 

Our UPLC-MS analysis for intracellular accumulation of PFASs revealed the highest intracellular concentration for PFOS. We conclude that the compound uptake by cells is higher for PFOS than PFOA. Unfortunately, we were not successful in detecting GenX using this method. The same effect was observed on HS-DFM imaging; the cellular uptake of compounds is PFOS > PFOA > GenX. This difference in cellular uptake and accumulation could be due to the difference in the passive permeability of cell membrane to compounds or due to differences in active transport through organic anion transporter polypeptides (OATPs), which have been found in almost all body tissues [82], and are responsible for the transport of endogenous molecules and drugs into the cells. This transport requires the formation of substrate–receptor complex. This substrate–receptor interaction requires at least a three point attachment involving three oxygen atoms in the substrate [83], thus making PFOS more likely to be transported into the cell than PFOA and GenX to impart cytotoxicity. However, this mechanism needs further investigation which is beyond the scope of this article. The compound uptake by cells and their potential to cause dysregulation in the cells’ normal mechanism was found to be in close agreement with our experiments for the mRNA expression. The higher the concentration of a compound inside the cells, the more likely it has the potential to interfere with the normal functioning of cells. 

## 5. Conclusions

We used the non-small cell lung carcinoma cell line (A549) to determine *in vitro* toxicity of three key PFAS compounds on epigenetic alterations, cell proliferation cycle, and apoptosis. We identified profound effect of these compounds on epigenetic regulators to possibly result in hypomethylation. We also noticed that lower doses of compounds might cause the activation of cell proliferation cycle, whereas higher doses may cause the balance to switch to apoptosis, following the CASP9–CASP3 axis. Experiments with mass spectrometry and dark-field microscopy indicated a higher uptake of PFOS by the cells. The observed dysregulation of the normal cellular proliferation and apoptosis could be a precursor to cancer development. Thus, exposure to these compounds may cause lung cancer, or lead to poor prognosis of pre-existing lung diseases. However, chronic exposure studies need to be further conducted in relation to cancer biomarkers and other disease-related markers. 

## Supplementary Materials

The following are available online at https://www.mdpi.com/2305-6304/8/4/112/s1, Figure S1: Hyperspectral images and spectral profiling of GenX (top row), PFOA (middle row) and PFOS (bottom row), Table S1: Sequences of primers used for quantitative real-time polymerase chain reaction (qRT-PCR). Table S1a: For epigenetic analysis; Table S1b: For cell cycle proliferation study. Table S1c: For apoptosis and its pathway studies.
