# Supplementary material for: Epigenetic Modifications, and Alterations in Cell Cycle and Apoptosis Pathway in A549 Lung Carcinoma Cell Line upon Exposure to Perfluoroalkyl Substances
Source: Toxics. 2020 Nov 23;8(4):112. doi: 10.3390/toxics8040112 (PMC7711517; doi:10.3390/toxics8040112)
Supplement: Supplementary file 1 [file toxics-08-00112-s001.pdf]

Supplementary Material: Epigenetic Modifications, and Alterations in Cell Cycle and Apoptosis Pathway in A549 Lung Carcinoma Cell Line upon Exposure to Perfluoroalkyl Substances

Musarrat Jabeen, Muhammad Fayyaz, and Joseph Irudayaraj

Table S1. Sequences of primers used for quantitative real-time polymerase chain reaction (qRT-PCR).

Target gene	Forward Primer	Reverse Primer
**GAPDH**	5′-GAA GGT GAA GGT CGG AGT C-3′	5′-GAA GAT GGT GAT GGG ATT TC-3′

Table S1a. For epigenetic analysis.

Target gene	Forward Primer	Reverse primer
**DNMT1**	5′-TAC CTG ACG ACC CTG ACC TC-3′	5′-CGT TGG CAT CAA AGA TGG ACA-3′
**DNMT3a**	5′-TAT TGA TGA GCG CAC AAG AGA GC-3′	5′-GGG TGT TCC AGG GTA ACA TTG AG-3′
**DNMT3b**	5′-GGC AAG TTC TCC GAG GTC TCT G-3′	5′-TGG TAC ATG GCT TTT GGA TAG GA-3′
**TET1**	5′-CAG AAC CTA AAC CAC CCG TG-3′	5′-TGC TTC GTA GCG CCA TTG TTA-3′
**TET2**	5′-GAT AGA ACC AAC CAT GTT GAG GG-3′	5′-TGG AGC TTT GTA GCC AGA GGT-3′
**TET3**	5′-TCC AGC AAC TCC TAG AAC TGA G-3′	5′-AGG CCG CTT GAA TAC TGA CTG-3′

Table S1b. For cell cycle proliferation study.

Target gene	Forward Primer	Reverse primer
**CCNE1**	5′-AAA TGG CCA AAA TCG ACA GG-3′	5′-CGA GGC TTG CAC GTT GAG TT-3′
**CCNA2**	5′-AGT AAA CAG CCT GCG TTC ACC-3′	5′-GAG GGA CCA ATG GTT TTC TGG-3′
**CCNB1**	5′-ATG ACA TGG TGC ACT TTC CTC C-3′	5′-GCC AGG TGC TGT ATA ACT GG-3′

Table S1c. For apoptosis and its pathway studies.

Target gene	Forward Primer	Reverse primer
**BAX**	5′-CAA ACT GGT GCT CAA GGC CC-3′	5′-GGG CGT CCC AAA GTA GGA GA-3′
**BCL-2**	5′-TAC CCA CTG AGA TTT CCA GGC-3′	5′-CAG GGC TTA AGG TAC TGG ATG A-3′
**BCL2L1**	5′-GCA GTA AAG CAA GCG CTG AGG-3′	5′-CAC AAT GCG ACC CCA GTT TAC C-3′
**CASP3**	5′-GGA AGC GAA TCA ATG GAC TCT G-3′	5′-CGA CAT CTG TAC CAG ACC GAG-3′
**CASP8**	5′-GCT TGT CAG GGG GAT AAC TAC C-3′	5′-CCA TCC CCA GCA GAA AGT CAG-3′
**CASP9**	5′-CAG TGG GCT CAC TCT GAA GAC C-3′	5′-GGG TGC AAG ATA AGG CAG GGT G-3′
**BID**	5′-GGT GTG AAG GTG ATT TAA GGG C-3′	5′-GCT ATT ACC AGG GGG CTA ACT C-3′

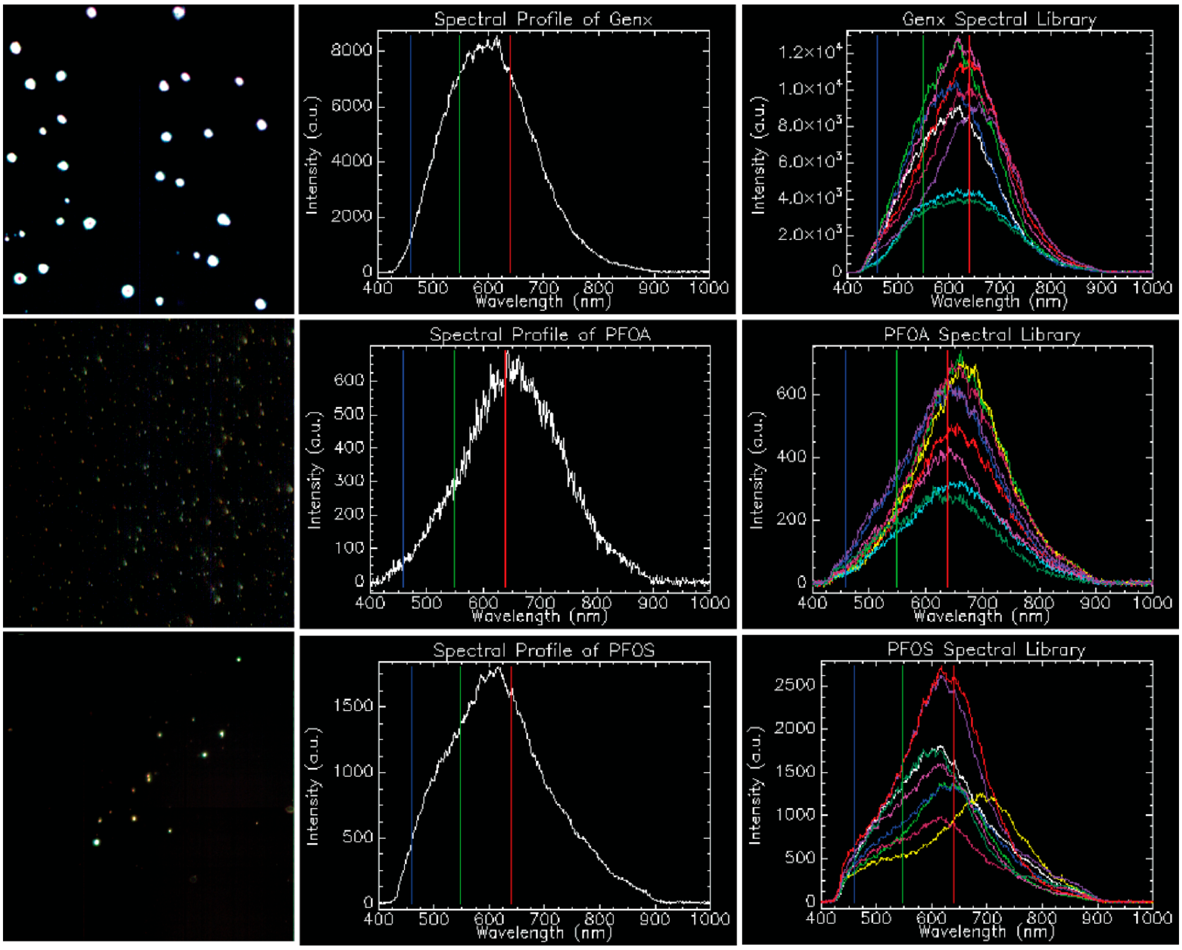

Figure S1. Hyperspectral images and spectral profiling of GenX (top row), PFOA (middle row) and PFOS (bottom row).
